# Brain Inhibitory Mechanisms Are Involved in the Processing of Sentential Negation, Regardless of Its Content. Evidence From EEG Theta and Beta Rhythms

**DOI:** 10.3389/fpsyg.2019.01782

**Published:** 2019-08-08

**Authors:** David Beltrán, Yurena Morera, Enrique García-Marco, Manuel de Vega

**Affiliations:** ^1^Instituto Universitario de Neurociencia, Universidad de La Laguna, San Cristóbal de La Laguna, Spain; ^2^Departamento de Psicología Cognitiva, Universidad de La Laguna, San Cristóbal de La Laguna, Spain; ^3^Centro Asociado de La Laguna, Universidad Nacional de Educación a Distancia, Madrid, Spain; ^4^Facultad de Psicología, Universidad Europea de Canarias, Santa Cruz de Tenerife, Spain

**Keywords:** sentential negation, two-step account, response inhibition, theta rhythms, beta rhythms, inhibition reuse

## Abstract

The two-step process account of negation understanding posits an initial representation of the negated events, followed by a representation of the actual state of events. On the other hand, behavioral and neurophysiological studies provided evidence that linguistic negation suppresses or reduces the activation of the negated events, contributing to shift attention to the actual state of events. However, the specific mechanism of this suppression is poorly known. Recently, based on the brain organization principle of neural reuse (Anderson, 2010), it has been proposed that understanding linguistic negation partially relies upon the neurophysiological mechanisms of response inhibition. Specifically, it was reported that negated action-related sentences modulate EEG signatures of response inhibition (de Vega et al., 2016; Beltrán et al., 2018). In the current EEG study, we ponder whether the reusing of response inhibition processes by negation is constrained to action-related contents or consists of a more general-purpose mechanism. To this end, we employed the same dual-task paradigm as in our prior study—a Go/NoGo task embedded into a sentence comprehension task—but this time including both action and non-action sentences. The results confirmed that the increase of theta power elicited by NoGo trials was modulated by negative sentences, compared to their affirmative counterparts, and this polarity effect was statistically similar for both action- and non-action-related sentences. Thus, a general-purpose inhibitory control mechanism, rather than one specific for action language, is likely operating in the comprehension of sentential negation to produce the transition between alternative representations.

## Introduction

Negation—as instantiated by operators like *not* and *no*—belongs to the special class of linguistic devices whose understanding in sentential contexts implies representing at least two different, often opposed alternatives. According to the so-called two-step process of negation (e.g., Kaup and Zwaan, 2003), negative sentences (e.g., *Today is not a bright day*) are semantically more complex than the corresponding affirmative sentences (e.g., *Today is a bright day*). The explanation is that the latter expresses only one idea, which corresponds to the actual state of affairs, whereas a negative sentence induces the reader/listener to represent the negated situation (e.g., *a bright day*) as well as the actual one (e.g., *a cloudy or dark day*). This conception is clearly supported by a recurrent finding reported in the literature: the comprehension of negative statements generally demands more cognitive resources and processing time than the comprehension of affirmative sentences (for reviews, Wason and Johnson-Laird, 1972; Kaup, 2001; Tian and Breheny, 2016; Papeo and de Vega, 2019).

An important aspect in the literature of the two-step account is that the representation of the negated events is temporary, since it is rapidly suppressed and replaced by the representation of the actual events. No doubt, managing two representations in negations (e.g., suppressing one and activating and keeping the other) requires efficient processes that often have been neglected. The present study tries to examine one of these processes, proposing that the response inhibition system could be responsible to make the transition between the initial and the actual representation derived from negative polarity sentences, by suppressing the former. Moreover, this paper posits that response inhibition is recruited for processing sentential negation, regardless of its content.

### From Representations to Processes

Cognitive research on negation has traditionally focused on the temporal dynamic of the two underlying representations, following the prevailing two-step process account. For instance, for the aforementioned negative sentence “Today is not a bright day,” this model proposes that a representation of the denied situation is activated first as if the negative operator had been removed, and hence creating a similar meaning representation as the affirmative counterpart (e.g., *Today is a bright day*). Next, in a second step, the negative operator starts to be integrated into the sentence meaning, resulting in deactivation of the initial representation, and a later replacement by the representation of the actual state of affairs (e.g., *A cloudy or dark day*). There are competing models to explain negation processing, and also empirical findings that question some of the assumptions of the two-step account, specially the one stipulating that the first step is mandatory (e.g., Mayo et al., 2004; Giora et al., 2007; Khemlani et al., 2012; Tian and Breheny, 2016). At least in some cases, sentential negation seems to be processed in the same way as affirmative sentences. For instance, world-knowledge violations in negative (e.g., *Zebras are not stripy*) and affirmative form (e.g., *Ladybirds are stripy*) induce the same N400 modulations and do not show any evidence of an additional processing step (Dudschig et al., 2018). Also, some studies described in the next section reported that negation induces a disembodiment effect in action language very early, as measured by grip force (Aravena et al., 2012) or corticospinal excitability measures (Papeo et al., 2016), suggesting a single-step processing of sentential negation.

In any case, all the theoretical and empirical approaches share a concern on what is represented and when, and also on how pragmatic factors—background knowledge and context information—modulate the whole process of sentence meaning comprehension (e.g., Beltrán et al., 2008; Nieuwland and Kuperberg, 2008; Dale and Duran, 2011; Orenes et al., 2014, 2016). However, negative and affirmative sentences differ not only in the number of alternatives (or representations) they invoke but also in the operations (or processes) recruited to manage these representations. We think that the analysis of these processes has been somehow neglected by previous studies, with a few recent exceptions (de Vega et al., 2016; Beltrán et al., 2018; Dudschig and Kaup, 2018).

Let us focus on the activation–inhibition processes proposed by the two-step account, which are inferred from the results obtained with experimental paradigms such as the probe recognition task (e.g., MacDonald and Just, 1989; Kaup, 2001; Kaup and Zwaan, 2003; Kaup et al., 2006). In this task, a sentence (or a short paragraph) is followed by a *probe* (a word or a picture), and participants have to recognize whether this *probe* was previously mentioned; in other versions of the task, participants simply name the probe aloud. The latency to the *probe* is the key measure, which is taken as an index of activation for the corresponding concept. A common pattern obtained was as follows: when the *probe* was shown shortly after the sentence, the time to recognize or name it was the same regardless of the polarity of the sentence (e.g., *The door is [not] open*), whereas when the interval between the sentence and the *probe* was large, then the recognition (or naming) latencies were larger for negative than for affirmative sentences (e.g., Kaup et al., 2006). The latter result is usually interpreted as reflecting the suppression or inhibition of the negated concept and hence as a demonstration of the second step for negation processing. Accordingly, one key feature of negation, relative to affirmative sentences, is that, over time, it recruits additional processes. But what are these additional processes? Based on the empirical consequences of negation, most researchers agree that the function of these processes is twofold: inhibiting (or suppressing) the negated content and updating (or activating) a representation of the actual situation. Still, they have not usually gone beyond this general description. Which is the brain machinery underlying these processes? Is it a neural network specifically involved in the syntactic processing of sentential negation? Or, by contrast, is it a general-purpose inhibitory control network, primarily involved in monitoring alternative actions and reused to managing alternative linguistic meanings in sentential negation?

As we will see in the next section, a first approach to the neural bases of understanding sentential negation derives from the embodiment research program, aimed to specify how sensory-motor systems contribute to represent sentence meaning. Specifically, several researchers applied the embodied approach to contrast affirmative and negative action sentences.

### Embodied Research on Negation

The embodied approach to language comprehension posits that meaning is grounded in the activity of non-linguistic systems (for recent reviews, Barsalou, 2016; García and Ibáñez, 2016). Crucially, there is extensive empirical evidence of embodied effects during the comprehension of action-related language, demonstrating that it partially relies on the activation of the motor mechanisms. For instance, the behavioral paradigm action-sentence compatibility effect (ACE) has shown that understanding action sentences interacts (facilitating or interfering) with performance in a concurrent matching motor task (e.g., Glenberg and Kaschak, 2002). Also, motor and premotor cortex processes associated with action language have been revealed by neuroimaging (e.g., Tettamanti et al., 2008; Moody and Gennari, 2010; Tomasino et al., 2010; de Vega et al., 2014), electroencephalography (e.g., Aravena et al., 2010; van Elk et al., 2010; Moreno et al., 2013, 2015), non-invasive brain stimulation (e.g., Buccino et al., 2005; Tomasino et al., 2008; Papeo et al., 2009), and brain-injured patient studies (e.g., Boulenger et al., 2008; Herrera et al., 2012).

The most remarkable phenomenon for the purpose of this article is that the presence of a negative operator in action-related statements produces a “disembodiment” effect by reducing motor activation, compared to their affirmative counterparts. Thus, behavioral studies have demonstrated that negation reduces peripheral motor activity underlying the semantics of action language (Aravena et al., 2012; Bartoli et al., 2013; Foroni and Semin, 2013). For instance, in Aravena et al.’s (2012) study, the participants kept in their right hand a grip force sensor while listening to affirmative or negative action sentences (*At the gym, Fiona lifts [doesn’t lift] the dumbbells*). The results showed that the grip force does not differ between affirmative and negative sentences during the first 200 ms after listening to the action verb. However, from this moment on, the grip force steadily increased until the end for affirmative action sentences, whereas it does not differ from baseline for negative action sentences. A recent study also reported that reading negated sentences referred to manual actions (e.g., *you don’t sign it*) interferes with typing the verb, whereas reading other negative statements referred to non-manual action (*you don’t talk to her*) or non-motor events (*you don’t believe it*) does not interfere with typing (García-Marco et al., 2019).

The effect of negation on manual action language has also been reported in some studies using neural measures. Thus, single-pulse TMS applied over the hand motor cortex revealed modulations in corticospinal excitability when reading affirmative manual verbs (*I write*), but not when reading negated manual verbs (*I don’t write*); by contrast, abstract verbs did not modulate motor excitability regardless of their polarity (*I wonder*/*I don’t wonder*) (Liuzza et al., 2011; Papeo et al., 2016; Experiment 1). Also, neuroimaging studies have shown increased activation of the motor and premotor cortex during the comprehension of affirmative action sentences and considerable reduction of these activations while understanding their negative counterparts (Tettamanti et al., 2008; Tomasino et al., 2010). Thus, overall, these investigations suggest that the motor system is recruited to process the meaning of affirmative action sentences, whereas it is deactivated or inhibited during the processing of negative action sentences.

Most of the above studies mainly reported disembodiment effects of negation in action-related linguistic contents, although specific effects of negation in non-action domains have also been obtained by Tettamanti et al. (2008), who reported a deactivation of the posterior cingulate cortex in negative abstract sentences compared to their affirmative counterparts. However, beyond the general or the content-specific neural deactivations induced by negation, none of the above studies proposed a general brain mechanism that could be responsible for these negation-induced deactivations. An interesting exception was a recent neuroimaging study, using a pattern analysis algorithm to reveal that affirmative and negative sentences regardless of their specific content differentially modulate the activation of several brain areas, including the left dorsolateral, the medial frontal cortex, the anterior and middle cingulate gyrus, and the precuneus (Ghio et al., 2018). However, in the same study the authors also found that negative sentences uniquely modulate content-specific brain areas for concrete sentences (left posterior temporal gyrus, left angular gyrus, right inferior frontal gyrus (IFG), and right superior frontal gyrus) and for abstract sentences (left temporal pole, right medial temporal lobe, right precuneus, and cerebellum), indicating that the impact of negation might be highly distributed and content-dependent.

### Neural Inhibition in Negated Action Sentences

Recently, it has been proposed that one of the neural mechanisms underlying the processing of negation is the response inhibition network of the brain (de Vega et al., 2016; Papeo et al., 2016; Beltrán et al., 2018). The inhibition system is a well-known network that includes prefrontal structures, such as the right IFG, and the pre-supplementary motor area (pre-SMA) among others, which are typically involved in inhibition and control processes observed in several experimental paradigms such as the Go–NoGo or the Stop signal (Aron et al., 2014, for a review). When the EEG is recorded during the performance of these tasks, response inhibition produces robust signatures. Thus, refraining from responding in NoGo trials, in the context of a prepotent response requested in the frequent Go trials, is associated with increased power in fronto-central theta band (4–7 Hz) rhythms (Nigbur et al., 2011; Huster et al., 2013; Harper et al., 2014), as well as enhanced amplitude of the N1, N2, or P3 components of the ERPs (Bokura et al., 2001; Maguire et al., 2010).

To explore how these inhibition signatures are modulated by sentential negation, de Vega et al. (2016) asked participants to read hand-action sentences with affirmative or negative polarity (i.e., *Now you will [will not] cut the bread*), with an embedded Go–NoGo task. As expected, the analysis of the EEG signal provided a strong increase of power in fronto-central theta rhythms in NoGo trials, compared to Go trials, indexing motor inhibition in the former. Crucially, this effect was qualified by the sentence polarity, given the fact that negative sentences diminished NoGo theta rhythms compared to affirmative sentences, whereas no effect of polarity was observed on Go trials. This Go/NoGo × polarity interaction suggests that response suppression and linguistic negation may share inhibitory mechanisms. In another EEG study, Beltrán et al. (2018) asked participants to read the same affirmative and negative action sentences, while performing a Stop Signal task (SST). In the typical SST procedure, participants receive a Go cue in every trial, but in some trials, after a variable delay, they also receive a Stop signal, indicating prompting the suppression of the underway response. The Stop–Signal delay (SSD) contingently varies from trial to trial so as to produce around 50% successful stops. An interaction was obtained between sentence polarity and performance in stop trials (success vs. failure) in the N1 component, an early signature of inhibition processes, consisting of larger amplitude for successful trials with negative sentences than for successful trials with affirmative sentences, whereas no polarity effect was found in unsuccessful trials. The source of these modulatory effects of polarity was the right IFG, a prominent region in the neural network of response inhibition (Aron et al., 2014). Convergently, the estimated stop-signal reaction time showed that participants were significantly faster at inhibiting responses in the context of affirmative sentences than in the context of negative sentences.

Finally, in the aforementioned study by Papeo et al. (2016, Experiment 2), the authors measured the motor silent period, a marker of activity in the GABAergic system, following stimulation of the motor cortex while contracting the right-hand muscles. They obtained larger silent period while processing negated action sentences, compared to their affirmative counterparts, concluding that negation not only reduces motor activity but also recruits inhibitory processes.

A complementary hypothesis of negation has been proposed by Dudschig and Kaup (2018), according to which negation would rely on conflict-monitoring processes to cope with the two alternative representations. In their experiment, they used an analog of the Simon task in which the participants had to press the requested right or left key in the keyboard, when reading affirmative (“now right” or “now left”) or negative (“not right” or “not left”) prompts. They recorded the ERP lateralized readiness motor potential (LRP) that allowed exploring the time course of the initial (counterfactual) representation of negative statements (e.g., “*left*” in “*not left*”) and the final (factual) representation (e.g., “*right*” in “*no left*”). Initially, the LRP corresponded to the counterfactual meaning (e.g., right hemisphere activation in “*not left*”), and later on it reversed indexing the factual representation (e.g., left hemisphere activation in “*not left*”). These results with a very specific type of linguistic negation clearly support the two-step process theory and, according to the authors, also indicate a conflict monitoring process similar to that reported in the studies with the Simon task.

The results of the above experiments go beyond the “disembodiment” effects of negation previously reported, demonstrating for the first time that linguistic negation consumes neural resources of response inhibition and/or conflict monitoring. However, the experiments just employed hand-action sentences and it is not clear whether inhibition is a general feature of negation or it is only recruited when negation is applied to action contents.

### The Current Research

**FIGURE 1:**
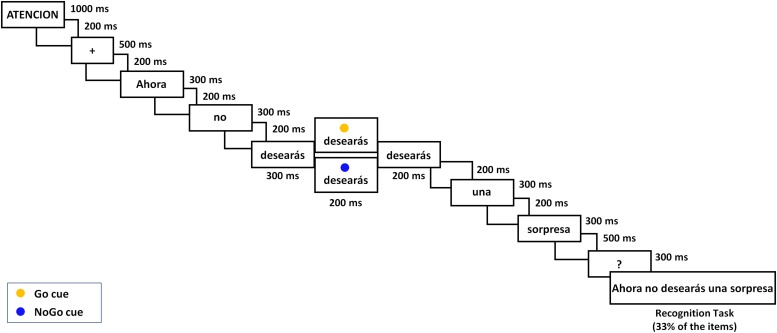
Outline of an experimental trial with a negative mental sentence (ATTENTION/Now/you will not/wish/any/surprise/?/Now you will not wish any surprise); 70% of trials received a Go cue (yellow circle) and 30% received a NoGo cue (blue circle).

This study deals with an important question that remains unanswered. Based on the reported evidence, let us assume the two-step account as a default hypothesis for the processing of sentential negation. Let us also accept that the neural mechanism of inhibitory control is involved to some extent in the processing of some negative sentences. Given these premises, does inhibition work locally just on the motor system and, consequently, does it exclusively support the processing of negated action language? Or, alternatively, is inhibitory control a general-purpose mechanism operating in the processing of all negative sentences? The only evidence of the latter was the aforementioned Ghio et al. (2018) study that reported that negation, independently of its semantic content, modulates a broad neural network, with “syntactic and cognitive control” functions. In this study, we go one step further to test the generality of control inhibitory processes in sentential negation, recording neurodynamical rather than neuroanatomical data, which provide specific signatures of inhibition with fine-grained temporal resolution. To this aim, we performed an EEG study with the same dual-task paradigm as the one employed by de Vega et al. (2016). Namely, participants read affirmative and negative sentences for comprehension, while simultaneously performing a Go–NoGo task, receiving the corresponding cue 300 ms after the verb onset and 800 ms after the appearance of the polarity marker (see Figure 1). Note that this timing implies that the impact of negation on response inhibition was examined quite early, in a stage in which the negative marker and the verb are being integrated and before completing the processing of the whole sentence. This could be a critical moment to register neural markers of the inhibitory control processes, which could be responsible either for suppressing the initial representation of the negated situation—in the two-step account—or for preventing its activation—in an incremental single-step view.

Time–frequency analysis was time-locked to the Go–NoGo cue, with a focus on modulations in fronto-central theta rhythms (4–7 Hz). We also analyzed modulations in the right-frontal beta rhythms (13–30 Hz), which can also be sensitive to inhibitory control processes according to some recent studies (Zhang et al., 2008; Klepp et al., 2015; Wagner et al., 2018). We expect that negative sentences, compared to affirmative ones, modulate these rhythms especially when they appear in the context of response inhibition (NoGo trials). The rationale of this prediction, already probed in de Vega et al.’s (2016) study, is that the ongoing processing of negative sentences interacts with the response suppression triggered by the NoGo cue, given the fact that both share neural resources of inhibitory control. Then, this interaction is expected to happen during the developing of the first processing step of negation, namely, during the activation of the mental representation for negated information.

Critically, and unlike in previous studies, we manipulated the linguistic content including both motor action sentences (e.g., *Now you will [will not] cut the bread*) and mental events sentences (e.g., *Now you will [will not] wish a surprise*). We expect to find increase in power of theta and beta rhythms for NoGo trials, but these inhibitory markers will be also modulated by negation. What is more important, we will be able to answer our main question. If the modulatory effects of negation on these neural signatures occur just for action language, then we will have a local content-specific recruitment of the inhibitory control system. Namely, the response inhibition network would only operate on the motor cortex and therefore would only modulate negative action language. This possibility exists, given the fact that many studies on sentential negation, registering motor performance or corticospinal excitability, found disembodiment effects for action language and null effects for non-action language (Aravena et al., 2012; Bartoli et al., 2013; Foroni and Semin, 2013). By contrast, if the modulatory effects of negation are shared by motor and mental contents, then we may support the hypothesis that inhibitory control processes are a general mechanism underlying sentential negation. Note, however, that even if theta rhythms were equally modulated by action and non-action negative sentences, this would not preclude the possibility that other content-specific networks are differentially affected by the negation marker, even though our EEG time–frequency analysis cannot dissociate them.

## Materials and Methods

### Participants

A total of 27 undergraduate students of psychology participated in this experiment (19 females; age range, 19–26 years old). All participants gave written informed consent and received course credit for their participation. All were neurologically healthy, right-handed native Spanish speakers and had normal or corrected-to-normal eyesight. The study was approved by the Ethics Committee of the University (Register CEIBA2014-0126, Comité de Ética de la Investigación y Bienestar Animal. Vicerrectorado de Investigación y Transferencia de Conocimiento. Universidad de La Laguna. 38071, La Laguna, Santa Cruz de Tenerife, Spain).

### Materials

A total of 532 five-word experimental sentences were created; 266 with motor action verbs (involving the use of hands) and 266 with mental verbs (involving cognitive or mental processes). The motor and the mental verbs were matched in frequency and length (see Supplementary Table 1), according to the EsPal database (Duchon et al., 2013), whereas, as expected, they differed in imageability, *t*(28) = 11.786, *p* < 0.001. Each verb appeared in 12 or 13 different sentences across the whole set of stimuli. Eighty additional filler sentences were also created, differing from the experimental ones in using different temporal adverbs and types of verbs. For each experimental sentence, there were two polarity versions: affirmative and negative. About one third of the sentences were followed by a recognition task to encourage participants to pay full attention to their meaning. This task consisted of a literal repetition of the previous sentence (response *yes*) or an altered version in which the polarity marker, the verb or the noun, differed from the original version (response *no*). Table 1 shows examples of materials.

**TABLE 1:** Examples of experimental and filler sentences (with literal translations into English in parentheses).

**Motor action:**
*Ahora sí [no] cortarás el pan (Now you will [will not] cut the bread)*
Possible control questions^*^
*Ahora sí cortarás el pan (Now you will cut the bread)*
*Ahora no cortarás el pan (Now you will not cut the bread)*
*Ahora sí comprarás el pan (Now you will buy the bread)*
*Ahora sí cortarás el queso (Now you will cut the cheese)*
**Mental action:**
*Ahora sí [no] desearás una sorpresa (Now you will [will not] wish a surprise)*
Possible control questions^*^
*Ahora sí desearás una sorpresa (Now you will wish a surprise)*
*Ahora no desearás una sorpresa (Now you will not wish a surprise)*
*Ahora sí prepararás una sorpresa (Now you will prepare a surprise)*
*Ahora sí desearás un consejo (Now you will wish an advice)*

^*^A control question followed the experimental sentences in one of four versions: correct for affirmative sentences, correct for negative sentences, incorrect verb, or incorrect noun.

### Design and Procedure

A repeated measure experimental design with 2 Cue (*Go*/*NoGo*) × 2 Polarity (*affirmative*/*negative*) × 2 Content (*motor*/*mental*) was employed. Each trial consisted of a sentence presented on a 24-inch monitor one word at a time, followed each by a blank screen; also, at a given moment, a Go or NoGo cue appeared over the sentence verb as Figure 1 illustrates. All events in a trial were controlled by means of E-prime software (version 2.1; Psychology Software Tools). Note that 300 ms after the verb onset, the Go/NoGo cue appeared above the word as a yellow or a blue circle, respectively, during 200 ms and the verb remained for an additional 200 ms (namely, a total of 700 ms). In *Go* trials (70%), in response to the yellow circle cue, participants should press with their right-hand index finger the letter “l” on the keyboard, which was covered with a yellow sticker. In *NoGo* trials (30%), cued by the blue circle, participants should refrain from pressing any key. One third of the trials were followed by a verification sentence that was presented 800 ms after the sentence last word. The verification task consisted of responding whether or not the sentence matched the previous one by pressing with left-hand middle or index finger one of two keys labeled as “yes” or “no,” respectively (corresponding to the 1 and 2 numbers in the upper left part of the keyboard). The verification sentences were correct in 50% of trials.

The structure of the session was as follows. First, the participants received instructions of the experiment followed by 16 practice trials. Thereafter, they were given six blocks of Go/NoGo trials. Four of these blocks included 101 trials each: 44 with affirmative, 44 with negative, and 13 with filler sentences; the other two blocks included 104 trials each; 45 with affirmative, with 45 negative, and 14 with filler sentences. The polarity of sentences was counterbalanced among participants, namely, a given content was presented as affirmative for half of the participants and as negative for the rest. Half of the participants began the experiment with a set of three blocks containing motor sentences (and fillers) followed by another set of three blocks including mental sentences (and fillers), and for the remaining participants, the order of the sets was reversed. Within each set, the blocks were randomly ordered for each participant, and within each block, the trial order was also randomized. The ratio of Go/NoGo trials (70%/30%) remained constant in all blocks of the experiment. The assignment of sentences to Go and NoGo trials was fixed (not counterbalanced), although the main lexical variables were matched for verbs (frequency and length) and nouns (frequency, length and imageability) in both kinds of trials, as Table 2 shows. The duration of the experiment was 1 h approximately. Correct response reaction times and accuracy were collected for both the *Go/NoGo* task and the verification task.

**TABLE 2:** Mean scores of lexical frequency, length (number of letters), and imageability of the verbs and the noun used in Go and NoGo trials.

	**Verb**		**Noun**	
	**Go**	**NoGo**	**Go**	**NoGo**
**Motor sentences**				
Frequency	0.93	0.93	0.85	0.91
Length	6.28	6.28	6.47	6.23
Imageability	5.37	5.37	6.03	6.09
**Mental sentences**				
Frequency	0.99	0.99	1.60	1.68
Length	6.57	6.57	7.33	7.37
Imageability	3.26	3.26	4.44	4.49

### EEG Recording and Pre-processing

EEG and EOG signals were recorded using Ag/AgCl electrodes mounted in elastic Quick-caps (Compumedics). EOG signal was measured from two bipolar channels: one from two electrodes placed at the outer canthus of each eye and the other from two electrodes above and below the left eye. EEG signal was recorded from 60 electrodes arranged according to the standard 10–20 system, with additional electrodes placed at cb1/cb2 and also on the left and right mastoids (M1/M2). All EEG electrodes were referenced online to an electrode at vertex and re-referenced offline to an average reference. EEG and EOG signals were amplified at 500 Hz sampling rate using Synamp2 amplifier (Neuroscan; Compumedics), with high- and low-pass filters set at 0.05 and 100 Hz, respectively. EEG electrode impedance was kept at <5 kΩ. EEG data preprocessing and analysis were conducted using Fieldtrip Toolbox (Oostenveld et al., 2011). Trial epochs were extracted from 2.5 s precue (Go/NoGo signal) onset to 2.5 s post cue onset, resulting in 5-s epochs. Trials with drifting or large movement artifacts were removed by visual inspection before analysis. Independent component analysis was applied to the data to remove the effects of blinks and eye movements. Remaining trials with EEG voltages exceeding 70 μV measured from peak to peak at any channel were also removed. After the application of the whole artifact correction–rejection procedure, a total of 12% of trials were rejected for the Go condition and 13% of trials for the NoGo condition.

### TFR Analysis

For the computation of the time–frequency representation (TFR), spectral power (1–30 Hz) was obtained by convolving 6-cycle complex Morlet wavelets with each single-trial EEG epoch. The resulting EEG power representations were normalized by subtracting, in a frequency fashion, the baseline from the power in every time point and dividing this difference by the baseline mean power. The 500 ms preceding the onset of the polarity word (affirmative “sí,” negative “no”) was used as the baseline, which means that resulting TFRs reflect power changes relative to this period. Finally, before the statistical analysis, the single-trial TFRs were averaged separately for each of the eight experimental conditions.

The resulting averaged TFRs were evaluated statistically using the cluster-based random permutation method implemented in Fieldtrip (Maris and Oostenveld, 2007). This method deals with the multiple comparisons in frequency, space, and time by identifying, over the whole ERP segment (here, 61,500 points: 15 frequencies, from 1 to 30 Hz in two-frequency step, 100 temporal points, and 60 electrodes), clusters of significant differences between conditions (sample points in close frequency, spatial and temporal proximity) while effectively controlling for type 1 error. This statistical approach allows only for pairwise comparisons. Therefore, certain prior calculations were performed to evaluate the current experimental design.

First, for the main effect of Cue, sentence Polarity and Content were collapsed for each participant and Cue condition, and then a cluster-based randomization comparison was conducted on the resulting Go and NoGo TFRs. This strategy allowed us to identify clusters with significant inhibition-related effects. Next, the identified clusters were submitted to subsequent analyses using the whole experimental design. More specifically, for each participant (*n* = 27) and condition (*n* = 8), a single power value was obtained by averaging the frequency, temporal, and spatial points that formed the inhibition-related cluster, and further submitted to a three-way, repeated measures ANOVA with Cue (Go, NoGo), Polarity (Affirmative, Negative), and Content (Motor, Mental) as within-subject factors.

## Results

### Behavioral Data

#### Go–NoGo Task

Table 3 shows the descriptive statistics for all the behavioral data. A Content (motor vs. mental) × Polarity (affirmative vs. negative) analysis of variance (ANOVA) was performed for Go reaction times (RT), after eliminating response errors (about 1.27%) and times exceeding 3 SD the individual mean (about 1.3%). The percentage of commission errors (in NoGo trials) and omission errors (in Go trials) were also computed and submitted to a Content × Polarity × Cue ANOVA. No significant effect was obtained for Go reaction times, *F*(1, 26) = 1.17, η^2^ = 0.043. There were more commission than omission errors, but the effect did not reach the significant threshold, *F*(1, 26) = 3.50, *p* = *0.07*, η^2^ = 0.119. All the other effects showed *F* and η^2^ values below 1.22 and 0.045, respectively.

**TABLE 3:** Behavioral data.

		**Motor**		**Mental**	
		**Polarity**		**Polarity**	
***Cue***		**Affirmative**	**Negative**	**Affirmative**	**Negative**
**Go/NoGo task**					
*Go*	RT	357 (8.9)	361 (9.4)	362 (9.7)	362 (9.1)
	ERR	0.01 (0.01)	0.01 (0.01)	0.01 (0.01)	0.01 (0.01)
*NoGo*	RT				
	ERR	0.02 (0.03)	0.02 (0.03)	0.02(0.04)	0.02 (0.03)
**Verification task**					
*Go*	RT	1442 (57.3)	1494 (60.1)	1492 (55.4)	1525 (54.0)
	ERR	0.03 (0.01)	0.05 (0.01)	0.03 (0.01)	0.07 (0.01)
*NoGo*	RT	1546 (66.5)	1576 (60.6)	1571 (66.9)	1573 (57.0)
	ERR	0.06 (0.01)	0.06 (0.01)	0.04 (0.01)	0.06 (0.02)

*Mean reaction times (RT) in milliseconds and error rates (0 to 1) in the Go/NoGo task and in the control task as a function of Content (motor vs. mental), Polarity (affirmative vs. negative), and Cue (Go vs. NoGo). The standard errors of the mean are shown in parentheses.*

#### Recognition Task

A Content × Polarity × Cue ANOVA was performed for response reaction times (RT), after eliminating response errors (about 1.7%) and times exceeding 3 SD the individual mean (about 3.7%). The main effect of Cue was significant, *F*(1, 26) = 38.68, *p* < 0.001, η^2^ = 0.598, as responses were faster when preceded by Go (*M* = 1,488 ms) than by NoGo trials (*M* = 1,566 ms). Also, responses were faster for affirmative than for negative sentences, although this effect did not reach the significant threshold, *F*(1, 26) = 2.94, *p* = *0.09*, η^2^ = 0.101. There was no other significant effect for recognition latencies. Concerning the analysis on the proportion of errors, there was a significant main effect of Polarity, *F*(1, 26) = 15.113, *p* < 0.001, η_p_^2^ = 0.36, resulting from larger amount of errors in negative (*M* = *0.06*) than affirmative (*M* = *0.04*) sentences. Both the main effect of Cue, *F*(1, 26) = 3.55, *p* = 0.07, η^2^ = 0.120, and the interaction between Cue and Polarity, *F*(1, 26) = 2.38, η^2^ = 0.084, failed to reach significance. The *F* and η^2^ values for all other effects were below 1.66 and 0.060, respectively.

### TFRs Results: Inhibition-Related (NoGo vs. Go) Clusters

The time–frequency decomposition showed the expected pattern of strong increases in low frequency power (peaking around theta range, 4–7 Hz) after the cue signal onset, relative to the baseline period—the 500 ms preceding affirmative and negative particle onset. Power increases were maximal in fronto-central sites and larger for NoGo than for Go trials. Though of a small size, there were also power decreases for frequencies in the beta range (from 13 to 30 Hz), which were maximal in posterior regions but still visible in frontal and central sites, and stronger for Go than for NoGo trials. These inhibition-related differences in theta and beta power were part of the same and large cluster identified using the cluster-based method for the comparison between NoGo and Go trials, *T*_maxsum_ = 10,020, *p* < 0.001. Thus, to better examine the dynamics within each frequency range, we conducted two additional cluster-based comparisons, one for the low-frequency (2–10 Hz) and another for the high-frequency (11–30 Hz) range, which we will describe below.

#### Theta Modulations

**FIGURE 2:**
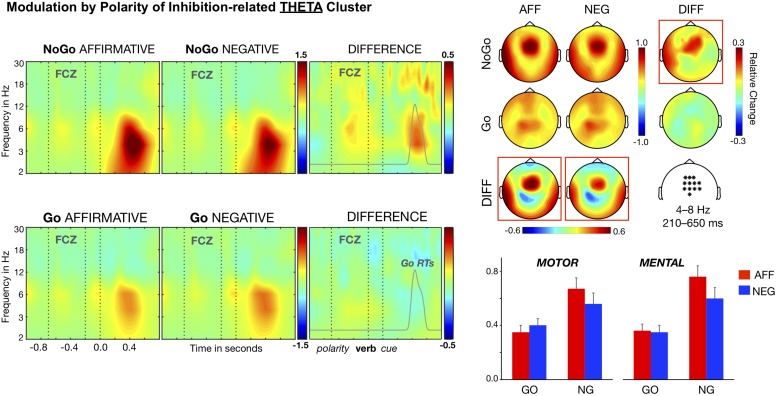
Time–frequency analysis of the Polarity × Cue interaction. Theta band clusters (6–8 Hz) averaged over the fronto-central electrodes (marked in the white map) are shown in the left side panel. A statistically significant cluster of polarity difference arises in NoGo trials. Affirmative-NoGo trials elicited larger theta power than negative-NoGo trials in the time window of 210–650 ms after the cue onset. This NoGo cluster corresponds to the response inhibition stage, since it overlaps the distribution of Go RTs (gray curve). The distributions of theta band modulations on the scalp are shown in the upper right panel of the figure. The bars in the lower right panel show that the differential polarity effects in NoGo trials for the theta band was similar in motor and mental contents.

The cluster-based comparison for the low-frequency range (2–10 Hz) identified stronger power increase, relative to baseline, for NoGo than for Go trials, from approximately 200 to 650 ms after the cue onset *T*_maxsum_ = 1284, *p* < 0.001. This cluster was maximal for the theta band (4–8 Hz) and located at medial sites of frontal and central regions. To explore the whole design in this inhibition-related cluster, a single cluster magnitude was computed, for each participant and condition, by averaging the amplitudes corresponding to the period (between 0.2 and 0.65 s), frequency (4–8 Hz), and topography showing maximal differences between NoGo and Go trials. We conducted next a Content (Motor, Mental) × Cue (Go, NoGo) × Polarity (Affirmative, Negative) ANOVA on this cluster magnitude. This analysis yielded the expected Cue main effect, *F*(1, 26) = 18.55, *p* < 0.001, η^2^ = 0.416, and most important the interaction Cue × Polarity, *F*(1, 26) = 9.76, *p* < 0.005, η^2^ = 0.273, but failed to produce main effects of Polarity, *F*(1, 26) = 3.49, η^2^ = 0.119, or Content, *F*(1, 26) < 1, η^2^ = 0.007, and of any interaction involving the Content Factor, *F*s (1, 26) < 1.76, η^2^ < 0.070. As Figure 2 shows, theta power increases for the NoGo trials were smaller in the context of negative (*M* = 0.58) than in the context of affirmative sentences (*M* = 0.70), *t*(26) = 3.07, *p* < 0.005, Cohen’s *d* = 0.592. In contrast, Go trials showed similar theta power magnitudes regardless of the polarity of the context, *t*(26) = 1.17, Cohen’s *d* = 0.225 (*M*s = 0.34 and 0.37 ms; *SE*s = 0.04). Moreover, although the inhibition-related effect—namely, stronger theta power increases for NoGo than Go trials—reached significance for the two polarity conditions, it was of a smaller size for the negative, *t*(26) = 2.98, *p* < 0.001, Cohen’s *d* = 0.574, than for the affirmative context, *t*(26) = 5.16, *p* < 0.001, Cohen’s *d* = 0.994. Thus, this pattern reflects minor increases in theta power for NoGo trials in the context of negative sentences, and hence confirms our prior findings (de Vega et al., 2016). Importantly, the factor Content had no effect on theta activity, which means that negation modulates theta band rhythms independently of the sentence content—either motor or mental.

#### Beta Modulations

**FIGURE 3:**
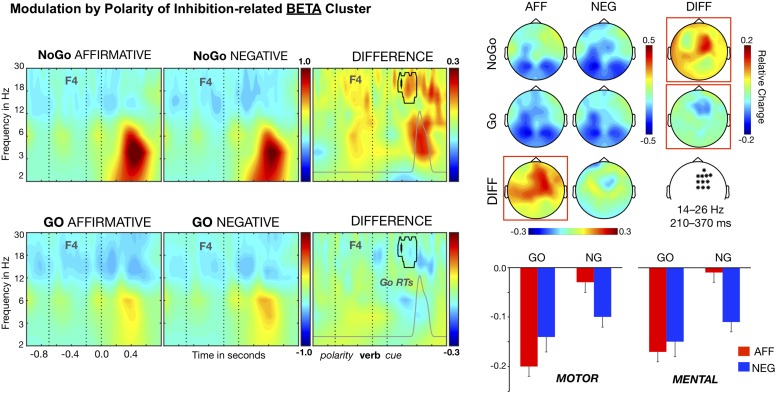
Time–frequency analysis of the Polarity × Cue interaction. Beta band clusters (14–26 Hz) averaged over the right fronto-central electrodes (marked in the white map) are shown in the left side panel. A significant differential sentence polarity cluster (surrounded by a black line) arises in both NoGo and Go trials. Affirmative-NoGo trials elicited larger theta power than negative-NoGo trials in the time window of 210–370 ms after the cue onset, partially overlapping the theta modulation and likely corresponding to the response inhibition stage (see Go RT distribution, signaled as the gray curve). The distributions of beta band modulations on the scalp are shown in the upper right panel. The bars in the lower right panel show that the differential polarity effects in both NoGo and Go trials for the beta band were similar in motor and mental contents.

As noted above, there was also a significant inhibition-related cluster in the higher frequency range (11–30 Hz), *T*_maxsum_ = 9133, *p* < *0.001*. This reflects a larger decrease in beta power, relative to baseline, for Go than for NoGo trials in a set of right fronto-central electrodes, for the period between 210 and 370 ms after the cue onset and for frequencies ranging from 14 to 22 Hz (see Figure 3). The subsequent three-way ANOVA yielded effects of Cue, *F*(1, 26) = 35.49, *p* < 0.001, η^2^ = 0.577, and the crucial interaction Cue × Polarity, *F*(1, 26) = 24.05, *p* < 0.001, η^2^ = 0.481, but failed to show significant effects of Polarity, *F*(1, 26) = 3.66, η^2^ = 0.124, Content, *F*(1, 26) < 1, η^2^ = 0.003, and the interactions involving the Content factor, *F*s (1, 26) < 2.40, η^2^ < 0.085. In the context of affirmative sentences, NoGo trials showed smaller beta power decreases than Go trials, *t*(26) = 8.26, *p* < 0.001, Cohen’s *d* = 1.59 (*M*s = −0.02 and −0.18; *SE*s = 0.02), whereas in the context of negative sentences, there was no significant difference between NoGo (*M* = −11) and Go trials (*M* = −14), *t*(26) = 1.68, Cohen’s *d* = 0.325. In addition, there were polarity effects for both Go and NoGo trials, but of opposite direction and distinct effect size. Hence, for Go trials, the power reduction was larger in the context of affirmative than in the context of negative polarity sentences, *t*(26) = 2.33, *p* = 0.023, Cohen’s *d* = 0.449, while the reverse happened for NoGo trials: stronger decreases in negative than affirmative polarity sentences, *t*(26) = 4.58, *p* < 0.001, Cohen’s *d* = 0.882. Thus, like for the theta power cluster, negative sentences modulated the inhibition-related effect, mainly by interfering with the reduction of beta power elicited by the NoGo trials.

## Discussion

Recently, it has been reported that negation modulates some neurophysiological markers of inhibition, suggesting that neural inhibition mechanisms could be involved in the processing of sentential negation (de Vega et al., 2016; Papeo et al., 2016; Beltrán et al., 2018; Liu et al., unpublished). However, most of these studies were limited to action-related sentences and their conclusions cannot be generalized to other linguistic domains. In contrast, the current study aimed to test the inhibition hypothesis of negation with two types of sentences, referring either to motor actions or to abstract events. Like in a previous study (de Vega et al., 2016), a Go–NoGo task embedded in the comprehension of affirmative and negative sentences was used but adding the manipulation of sentence semantic content. As expected in inhibition trials (NoGo), the increase in fronto-central theta power—a well-known marker of inhibition processes—was larger in the context of affirmative than in the context of negative sentences, confirming previous results in the literature (de Vega et al., 2016). Most important, this interaction between polarity and response inhibition happened regardless of the type of negated content, suggesting that the response inhibition network operates as a content-free mechanism involved in the processing of negation.

de Vega et al. (2016) also reported a cue × polarity interactive modulation on delta rhythms (1 to 4 Hz) in their experiment 1, indexing a delayed post-response evaluation processes in Go trials. We did not replicate this delta modulation because the timing of the critical events in our trials (verb and cue presentation) was considerably faster in our study than in de Vega et al.’s experiment 1 and the Go theta effects require larger presentation times to emerge. Consistently, de Vega et al.’s experiment 2 employed the same event timing in trials as the current study and also did not find delta modulation. By contrast, we found a cue × polarity interaction on beta power oscillations over right fronto-central sites, which slightly precedes and overlaps the fronto-central theta effect, and therefore could be indexing the same inhibitory processes. In fact, fronto-central beta is also an accepted marker of response inhibition as reported elsewhere (Zhang et al., 2008; Huster et al., 2013; Wagner et al., 2018). The cue × polarity interaction on the beta band was driven by the strong differences in power between affirmative and negative sentences, especially in the context of inhibition (NoGo) trials. Again, there were no differential effects between the motor and the mental content on beta rhythm modulations, supporting the involvement of content-free inhibition associated with processing of sentential negation.

Concerning the behavioral measures, performance in the Go/NoGo task was characterized by a virtual ceiling effect, such that behavioral measures (Go reaction times and errors) were not sensitive to either the type of trial or the polarity of the sentence. Just like in the previous study (de Vega et al., 2016), the high accuracy rate in the current dual-task paradigm is likely due to the long inter-trial intervals between consecutive Go/NoGo cues, which precluded the setting of a very strong tendency to respond (e.g., Zamorano et al., 2014). Similarly, behavioral results confirmed the long-term effects of both cue and polarity on the sentence recognition task, by showing slower reactions for NoGo than Go trials, and higher error rates for negative than affirmative sentences; nonetheless, there was no significant interaction between the two factors, or of any of them with the type of negated content.

### Theta and Beta Modulations

The increase in fronto-central theta band rhythms has been associated with inhibition-related processes in response inhibition tasks (e.g., Huster et al., 2013), and in this sense, our finding of content-free modulation by negation is consistent with the interpretation we advanced in previous studies (de Vega et al., 2016; Beltrán et al., 2018): that linguistic negation and response inhibition share inhibitory resources. Critically, the modulation by polarity of the inhibition effect over right-frontal beta power adds supporting evidence to this interpretation. Several studies, especially those using the SST, have already described that inhibition modulates oscillations in the beta band (e.g., Zhang et al., 2008; Wagner et al., 2018). More specifically, transient increases—i.e., synchronization—have been observed following the onset of the stop signal, which are either absent or reduced for non-inhibition (Go) and failed inhibition (stop) trials (e.g., Huster et al., 2013; Wagner et al., 2018). Furthermore, the use of electrocortical (ECoG) recordings indicates that beta synchronization originated at cortical areas around the right inferior frontal cortex (rIFC), an area strongly associated with the implementation of response inhibition (e.g., Swann et al., 2012; Aron et al., 2014). Our right-frontal beta effect shows the same pattern of differences between inhibition (NoGo trials) and non-inhibition trials (Go trials), as well as a similar distribution on the scalp, and therefore could be also interpreted as reflecting inhibition-related processes, which are modulated by sentence polarity.

### The Generality of the Inhibitory Mechanism

The most important finding in the current study is that theinteraction between negation and response inhibition signatures—i.e., fronto-central theta and right-frontal beta power—is equally modulated by motor and mental sentences. This finding considerably reinforces the hypothesis that the suppression effects of negation reported elsewhere (MacDonald and Just, 1989; Kaup and Zwaan, 2003; Kaup et al., 2006; Aravena et al., 2012; Bartoli et al., 2013) may be the consequence of applying a multipurpose inhibition mechanism to internal representations (de Vega et al., 2016; Beltrán et al., 2018; Papeo and de Vega, 2019). This proposal takes benefit from the idea of neural reuse, which holds that evolutionarily ancient mechanisms are redeployed to implement more recently acquired functions, while keeping the primary function (Anderson, 2010). This evolutionary strategy seems preferable because of being biologically less costly than developing *de novo* brain circuits. Our previous findings indicated that negation shares inhibitory mechanisms with response inhibition; however, they were limited regarding the generalizability of the effects, as they were obtained by combining a motor task (response inhibition in Go/NoGo or SST) with the comprehension of motor sentences. The current study extends these findings by probing that this interaction is not restricted to the negation of motor concepts, but it likely occurs regardless of the semantic modality of negated concepts. Thus, the reusing of inhibition networks—as indexed by modulations of some of its oscillatory markers—is a general characteristic of linguistic negation. However, note that the observed effect of negation, which is shared by action and non-action sentences, does not rule out that content-specific networks may also be affected differentially by the negation operator (Ghio et al., 2018). The general machinery of inhibitory control (indexed by our theta and beta modulations) could impact specific sensory-motor and semantic networks associated with particular contents, through specific cortico-cortical connections.

### Inhibition and Control Monitoring

The embodied approach to language has highlighted what seems to be a clear case of neural reusing: the recruitment of action and perception brain systems for conceptual representations of meaning (e.g., for a review, Barsalou, 2016). However, the proper meaning of the negation markers, like the meaning of other grammatical and morphological elements in language, seems hard to ground on perceptual and motor systems. Our proposal offers an alternative way to account for negation processing from an embodied perspective; one in which negative operators recruit the processing systems involved in the regulation of other neural systems, including those of perceptions and actions. In other words, negation relies upon domain-general cognitive inhibition and/or control processes (de Vega et al., 2016; Dudschig and Kaup, 2018; Papeo and de Vega, 2019).

It is worth noting that response inhibition and control monitoring are two processes that, although related, could be functionally separated. In this sense, the modulation of theta oscillations has been clearly associated with response inhibition processes, involved in NoGo trials (in Go/NoGo tasks) or successful stop (in SST), and therefore could be considered a marker of neural inhibition (e.g., Huster et al., 2013). However, modulations in theta oscillations with source in medial prefrontal regions have also been reported in a variety of cognitive control tasks such as the Simon task or the flankers task, which demand conflict resolution and decision making rather than response inhibition (e.g., Nigbur et al., 2011; Cohen, 2014). The morphological and biological features of prefrontal neurons support oscillations in theta band, which could be associated with diverse high-order cognitive processes implemented in the same or neighbor populations of neurons in the prefrontal cortex (Cohen, 2014; Dippel et al., 2017). Accordingly, the finding of a modulation of fronto-central theta oscillations by negation is ambiguous, since it does not clearly specify whether it indexes response inhibition, control monitoring or both. In any case, the fact that negative sentences modulate theta oscillations in NoGo trials strongly supports that the mechanisms underlying response inhibition are involved to some extent in the semantics of negation.

Concerning the right fronto-central beta oscillations, it has been reported that they are selectively modulated in response inhibition tasks, supporting the claim that they constitute a genuine neurobiological marker of inhibitory processes (e.g., Zhang et al., 2008; Huster et al., 2013; Wagner et al., 2018). Consequently, the fact that right fronto-central beta oscillations are modulated by negation, especially in NoGo trials, could support a more specific interpretation of our results: sentential negation interacts with response inhibition processes, at least in the context of the Go–NoGo task. It remains to be tested whether similar modulations of beta oscillations rise for non-motor (cognitive) inhibitions. Our proposal is that negation conveys the recruitment of domain-general inhibitory control mechanisms, and hence it should interact with a variety of inhibition paradigms.

Note, however, that it could be possible that processing sentential negation recruits the two mentioned control mechanisms—response inhibition and conflict monitoring—at different moments (e.g., Giora et al., 2007; Orenes et al., 2014; Dudschig and Kaup, 2018). The two-step process assumes that negated information is activated first (e.g., *open door* for the sentence “*The door is not open*”) and immediately followed by the updating of the alternative representation (e.g., *closed door*), which corresponds to the actual state of affairs (e.g., Kaup et al., 2006; Giora et al., 2007; Nieuwland and Kuperberg, 2008; Orenes et al., 2014; Tian and Breheny, 2016). The initial activation process is thought to be automatic, governed by memory-based associative operations, and very similar to that involved in the processing of affirmative sentences (Deutsch et al., 2006, 2009). By contrast, more controlled, rule-based processes are thought to intervene in the second step, inducing a change of the initial representation (Deutsch et al., 2009; Dudschig et al., 2019). Therefore, dealing with two opposing representations—the negated and the actual state of affairs—seems to demand to some extent conflict monitoring and selection of alternatives, followed by suppression or inhibition of the initial alternative (Dudschig and Kaup, 2018). Indeed, there is an interesting parallelism with the processing sequence involved in ordinary response inhibition tasks. In both the Go/NoGo and the SST, a strong tendency to respond is created, which conflicts with the inhibition cues (NoGo and Stop, respectively). This implies that, in inhibition trials, an initial step of response activation is followed by conflict detection—triggered by NoGo or Stop cues—and by the selection of an alternative course of action (inhibition plus updating).

Nonetheless, the current study probably gives an incomplete view of the neural dynamics in sentential negation processing. The interactive effects of negation on theta and beta rhythms were observed in a relatively early temporal snapshot, while the negation and the verb were still being integrated and at the time of the Go/NoGo cue presentation (this was also the case in previous studies with inhibition task paradigms: de Vega et al., 2016; Beltrán et al., 2018). At first sight, this timing seems inconsistent with the hypothetical two steps involved in the processing of negation. According to the two-step’s proposal, the negation should not modulate the neural activity as early as we have found because the polarity marker supposedly does not initially affect the first-step representation. Even more, these early interactive effects could be compatible with a single-step model of negation, in which, for instance, inhibitory control processes could operate incrementally from the beginning precisely to impede the activation of the negated situation model. This might be especially plausible when the previous linguistic or pragmatic context biases the actual meaning of the negated sentence. However, it is also possible that this early interaction between inhibition and negation is reflecting neural processes that were not detected by previous studies reporting a late impact of negation on behavioral or ERP measures (e.g., for a recent review, Kaup and Dudschig, 2019). Most prior behavioral and ERP studies have mainly focused on detecting the representational states associated with negation by measuring indexes of semantic processing such as the N400 component in world-knowledge or semantic violation sentences (e.g., Fischler et al., 1983; Nieuwland and Kuperberg, 2008; Dudschig et al., 2018, 2019) or reaction times in probe recognition tasks (MacDonald and Just, 1989; Kaup, 2001; Kaup and Zwaan, 2003), neglecting the conflict monitoring and inhibition processes that underlie the two-step process dynamics. By contrast, the time–frequency analysis of the EEG in the context of the dual-task paradigm we employed here may reveal an early operation of the inhibitory control mechanism that governs the transition from the initial to the final representation of the negated events (Dudschig et al., 2019) or prevents the representation of the negated situation. Interesting issues for further research are the extent to which the early inhibitory control process is automatically activated by negation operators, and the role that pragmatics (e.g., the pre-activation of negated meaning by the preceding context) plays in the initiation of conflict monitoring and the inhibition processes associated with negation.

This study has some limitations that must be overcome in further research. First, although the modulation of theta and beta signatures by negation was strong and content-free, we may note that it was obtained in a dual-task paradigm. Namely, the modulatory effects only emerged in the context of inhibitory NoGo trials. Future experiments, with alternative techniques (neuroimaging, TMS, electrophysiological functional localizers), will be needed to obtain differential neurobiological signatures of inhibition and control during the comprehension of sentences differing in polarity, without performing any other parallel task. Second, although the study made an important step in generalizing the hypothesis of neural inhibition, negation has many semantic and pragmatic dimensions, and the generality of the hypothesis needs additional proofs employing more diverse contents, such as perceptual, existential, or emotional. Third, the imperative format of our negative sentences can be partially responsible for the observed effects, given the fact that imperatives are functionally equivalent to a stop signal (de Vega et al., 2016). Additional studies are needed, using purely declarative sentential negations to determine whether inhibitory control processes also underlie other syntactic structures. Fourth, a more careful evaluation is needed to explore the relative role played by inhibition and control monitoring mechanisms during the processing of the two alternative meanings of negation. Fifth, our recording of interactive effects was constrained to an early time window associated with the verb, and other possible effects beyond this point were not registered in the study. In principle, integrative processes of negation considerably extend in time and we did not exhaust the analysis of potentially relevant effects, for instance of the content. What we registered was the early impact of the general control inhibitory mechanisms in negation, which could spread out to content-specific networks in a later stage. In other words, this inhibitory control mechanism may govern the deactivation of content-specific networks, such as the motor system, the visual system or the semantic hubs for action, visual or abstract sentences, respectively. The study of the functional links between the general inhibitory system and the content-specific networks during the processing of sentential negation is a relevant topic for further research.

## Conclusion

In this article, we obtained two convergent electrophysiological signatures (theta and beta oscillations) confirming that sentential negation may share neural processes with response inhibition. We propose that this inhibitory mechanism contributes to fill a gap in the current models of negation processing. The two-step account proposes dynamic changes during the processing of negation: from an initial representation of the negated state of affairs to its suppression and replacement by a representation of the actual state of affairs. The incremental single-stage model posits that the representation of the factual meaning of negation is immediately activated, while the alternative (negated situation) representation is blocked. However, none of these models provide any mechanism responsibly for governing the suppression or blocking of the negated situation. We propose that our data support the idea that the neural network of inhibition is a plausible mechanism, either to impede the activation of the negated events representation (in the single-step model) or to produce the transition between the initial (negated events) representation and the final (current events) representation in the two-step process. This mechanism works in a content-free manner, given the fact that the same neurobiological markers were equally sensitive to negative motor sentences and to negative mental sentences.

## Ethics Statement

The studies involving human participants were reviewed and approved by the Comité de Ética de la Investigación y Bienestar Animal, Vicerrectorado de Investigación y Transferencia de Conocimiento, Universidad de La Laguna. The patients/participants provided their written informed consent to participate in this study.

## Author Contributions

DB: experimental design, data analysis, and manuscript writing. YM: linguistic materials, experimental script data collection, and manuscript writing. EG-M: data collection and manuscript writing. MdV: experimental design, experimental materials, planning of data analysis, and manuscript writing.

## Conflict of Interest Statement

The authors declare that the research was conducted in the absence of any commercial or financial relationships that could be construed as a potential conflict of interest.
