# Supplementary material for: Brain Inhibitory Mechanisms Are Involved in the Processing of Sentential Negation, Regardless of Its Content. Evidence From EEG Theta and Beta Rhythms
Source: Front Psychol. 2019 Aug 8;10:1782. doi: 10.3389/fpsyg.2019.01782 (PMC6694754; doi:10.3389/fpsyg.2019.01782)
Supplement: Supplementary file 1 [file Table_1.DOCX]

Lexical frequency, length (number of letters) and imageability of the action and mental verbs used in the experimental sentences (with English translation in parentheses).

| *Motor verbs* | *Frequency* | *Length* | *Imageability* | *Mental verbs* | *Frequency* | *Length* | *Imageability* |
| --- | --- | --- | --- | --- | --- | --- | --- |
| *Abrir (to open)* | *1.7* | *5* | *5.31* | *Admitir (to admit)* | *1.25* | *7* | *2.89* |
| *Amarrar (to tie)* | *0.21* | *7* | *5.62* | *Ansiar (to yearn)* | *0.05* | *6* | *3.00* |
| *Apretar (to tight)* | *0.58* | *7* | *4.93* | *Anular (to cancel)* | *0.88* | *6* | *3.00* |
| *Cerrar (to close)* | *1.5* | *6* | *5.51* | *Apoyar (to support)* | *1.76* | *6* | *4.20* |
| *Coger (to take)* | *1.09* | *5* | *4.28* | *Confiar (to trust)* | *1.10* | *7* | *2.93* |
| *Colgar (to hang)* | *0.58* | *6* | *5.77* | *Creer (to believe)* | *1.68* | *5* | *4.11* |
| *Copiar (to copy)* | *0.66* | *6* | *4.75* | *Deducir (to deduct)* | *0.90* | *7* | *2.83* |
| *Cortar (to cut)* | *1.18* | *6* | *5.71* | *Desear (to wish)* | *1.36* | *6* | *3.38* |
| *Doblar (to bend)* | *0.7* | *6* | *6.23* | *Dudar (to doubt)* | *1.00* | *5* | *2.98* |
| *Encender (turn on)* | *0.8* | *8* | *5.27* | *Evocar (to evoke)* | *0.57* | *6* | *1.85* |
| *Fregar (to scrub)* | *0.18* | *6* | *5.59* | *Idear (to devise)* | *0.34* | *5* | *2.61* |
| *Guardar (to keep)* | *1.26* | *7* | *4.69* | *Ignorar (to ignore)* | *0.94* | *7* | *3.21* |
| *Levantar (to lift up)* | *1.38* | *8* | *4.35* | *Imaginar (imagine)* | *1.23* | *8* | *4.24* |
| *Limpiar (to clean)* | *0.92* | *7* | *5.09* | *Intuir (to intuit)* | *0.33* | *6* | *2.88* |
| *Mover (to move)* | *1.24* | *5* | *4.73* | *Necesitar (to need)* | *0.96* | *9* | *3.96* |
| *Pelar (to peel)* | *0.57* | *5* | *6.15* | *Olvidar (to forget)* | *1.55* | *7* | *3.59* |
| *Planchar (to iron)* | *0.15* | *8* | *6.46* | *Pensar (to think)* | *2.01* | *6* | *4.67* |
| *Regar (to water)* | *0.43* | *5* | *6.38* | *Planear (to plan)* | *0.56* | *7* | *3.77* |
| *Servir (to serve)* | *1.67* | *6* | *5.36* | *Razonar (to reason)* | *0.59* | *7* | *3.07* |
| *Tirar (to throw)* | *1.09* | *5* | *5.26* | *Tolerar (to tolerate)* | *0.77* | *7* | *2.15* |
| *Tocar (to play)* | *1.56* | *5* | *5.42* | *Valorar (appreciate)* | *1.05* | *7* | *3.23* |
| *Means (SD)* | *0.93 (0.49)* | *6.14 (1.06)* | *5.37 (0.62)* |  | *0.99 (0.47)* | *6.52 (0.98)* | *3.26 (0.70)* |
